# First Observation of a Spontaneously Matured Female European Eel (*Anguilla anguilla*)

**DOI:** 10.1038/s41598-020-59331-6

**Published:** 2020-02-11

**Authors:** A. P. Palstra, P. Jéhannet, W. Swinkels, L. T. N. Heinsbroek, P. M. Lokman, S. Vesala, J. Tulonen, T. Lakka, S. Saukkonen

**Affiliations:** 1 Wageningen University & Research Animal Breeding and Genomics, Wageningen Livestock Research, PO Box 338, 6700 AH Wageningen, The Netherlands; 2 DUPAN Foundation, Bronland 12-D, 6700 AE Wageningen, The Netherlands; 3 Wageningen Eel Reproduction Experts B.V., Mennonietenweg 13, 6702 AB Wageningen, The Netherlands; 4 Department of Zoology, University of Otago, 340 Great King Street, PO Box 56, Dunedin, 9054 New Zealand; 5 Natural Resources Institute Finland (Luke), Survontie 9A, 40500 Jyväskylä, Finland; 6 Kotka Maretarium OY, Sapokankatu 2, 48100 Kotka, Finland

**Keywords:** Physiology, Zoology

## Abstract

This study reports on the first observation of a spontaneously matured female European eel. The 43-year-old eel, together with eleven other females, resided at an aquarium house since their capture in 2002 and stocking as glass eels in 1978. In June 2019, the girth of the belly of the female increased as a sign of oocyte maturation. The specimen had an estimated gonadosomatic index (GSI) of 47, only half of the oocytes were hydrated and matured, indicating that European eels are polycyclic batch spawners. The live eels of the cohort were still in the previtellogenic phase but their eye sizes were close to that of the matured eel. We hypothesize that substances released by other maturing and spawning fishes may have triggered puberty of the eel. This first observation, and the possibility of more eels maturing in the near future, provides a natural reference for the sexual maturation of the European eel.

## Introduction

Eels of the genus *Anguilla* are characterized by a catadromous migration from continental fresh and brackish water habitats to, often remote, oceanic spawning grounds. Temperate eels swim the longest distances^1^, the European eel being the most extreme example, covering 5–6000 km to the Sargasso Sea^2^ in the northwest Atlantic Ocean. Eels exhibit semelparity and probably die soon after one or few subsequent spawning events in one spawning season^3^. In captivity, when the reproductive migration is prevented, eels do not spontaneously mature. Long-term hormonal stimulation by hypophysation is commonly practiced to mature eels artificially^4^. Until now, this has offered the only opportunity to observe fully mature European eels.

In this short article, we report on the first spontaneously matured European eel ever observed. This specimen, a 43-year-old female, was stocked in the Finnish Lake Vesijärvi as a glass eel from France in 1978. This eel was part of a cohort of twelve very large female eels that was re-captured in 2002 and that was then transferred to the Maretarium in Kotka, Finland. On June 12 2019, the girth of the eel’s belly had increased to reflect oocyte hydration that occurs during the final stage of sexual maturation. One month later, on July 13, the female died without having released any eggs. Other eels of the same cohort displayed large eyes and some ate little food, if anything at all – these are possible signs of commencement of puberty and hold promise of more eels maturing in the near future.

## Methods

### Aquarium environment

In 2002, the eels were introduced into a 600 m^3^ tank, but one year later they were transferred to a smaller 8.1 m^3^ tank. Both tanks received Baltic seawater that fluctuated over the season in temperature (8–18 °C), salinity (2.33–4.53 ppt), pH (7.1–8.5) and colour (15–35 mg Pt l^−1^). Specifically, water temperatures in the period January 1 2019 until the moment of death of the matured female on July 13 2019 increased from 8.4 to 16.9 °C with two small peaks occurring in March (supplemental material). The light regime depended on opening hours: 9.30–17.00 h (and 11.30–19.00 h on Wednesdays) from January to June and from September to January, and 9.30–19.00 from June to September. Sand and UV filtration were applied. Eels were fed Baltic herring (*Clupea harengus membras*; 70%) and sprat (*Sprattus sprattus*; 30%) but since February 2019 also shrimp (*Pandalus borealis*) and blue mussel (*Mytilus chilensis*). In the 8.1 m^3^ tank, the eels were kept together with pike (*Esox lucius*). Importantly, in February 2019, brown trout (*Salmo trutta*), common roach (*Rutilus rutilus*) and bream (*Abramis brama*) were introduced into the tank. Bream displayed a spawning rash ever since the introduction, but they never spawned. The tank was connected to the same recirculating aquaculture system as other tanks in which European perch (*Perca fluviatilis*), pike-perch (*Sander lucioperca*), Eurasian minnow (*Phoxinus phoxinus*), round goby (*Neogobius melanostomus*) and also river lamprey (*Lampetra fluviatilis*) spawned during the spring of 2019.

### Measurements on the matured eel

When the eel had just died, on July 13 2019, she was weighed and photos were taken of an ovarian sample. The specimen was stored in the freezer and defrosted just prior to analysis on October 28 2019; the specimen was photographed, weighed and measured (total length, girth, eye diameters and pectoral fin length). Measures were used for calculations of the Fulton condition factor K, the eye index (EI^5^), body girth index (BGI^6^) and pectoral fin index (PFI^7^). Following dissection, ovary, liver, intestine and heart weights were determined to estimate somatic indices (organ weight relative to body weight times 100%), *i.e*., gonadosomatic index (GSI), hepatosomatic index (HSI), intestinal somatic index (ISI) and the cardiac somatic index (CSI). The swimbladder was examined for the presence of the swimbladder parasite *Anguillicoloides crassus* and otoliths were retrieved for age confirmation. One of the otoliths was grinded and etched with mild HCl dilution before dyeing in Neutral Red solution to amplify the visibility of the annuli.

### Measurements on live eels

The live eels from the same cohort were anaesthetised (benzocaine, 80 ppm), weighed and measured as described above, and subjected to ultrasound examination for non-invasive estimation of GSI values^8^. Husbandry conditions (light/dark regime; water temperature, salinity, pH and colour; broodstock feeds and presence of other fish species maturing and reproducing) had been monitored and were considered as potential triggers of sexual maturation.

### Ethics

The manipulations on live European eels were approved by the Dutch Central Committee for Animal Experimentation (CCD nr. AVD401002017817) and the Animal Experimental Committee of Wageningen University (IvD nr. 2017.D.0007.003) and were in keeping with the conditions in research permit STK543A, ESLH-2001–08613/Ym-21 issued to Kotka Maretarium Oy.

## Results

### The matured eel

**Figure 1:**
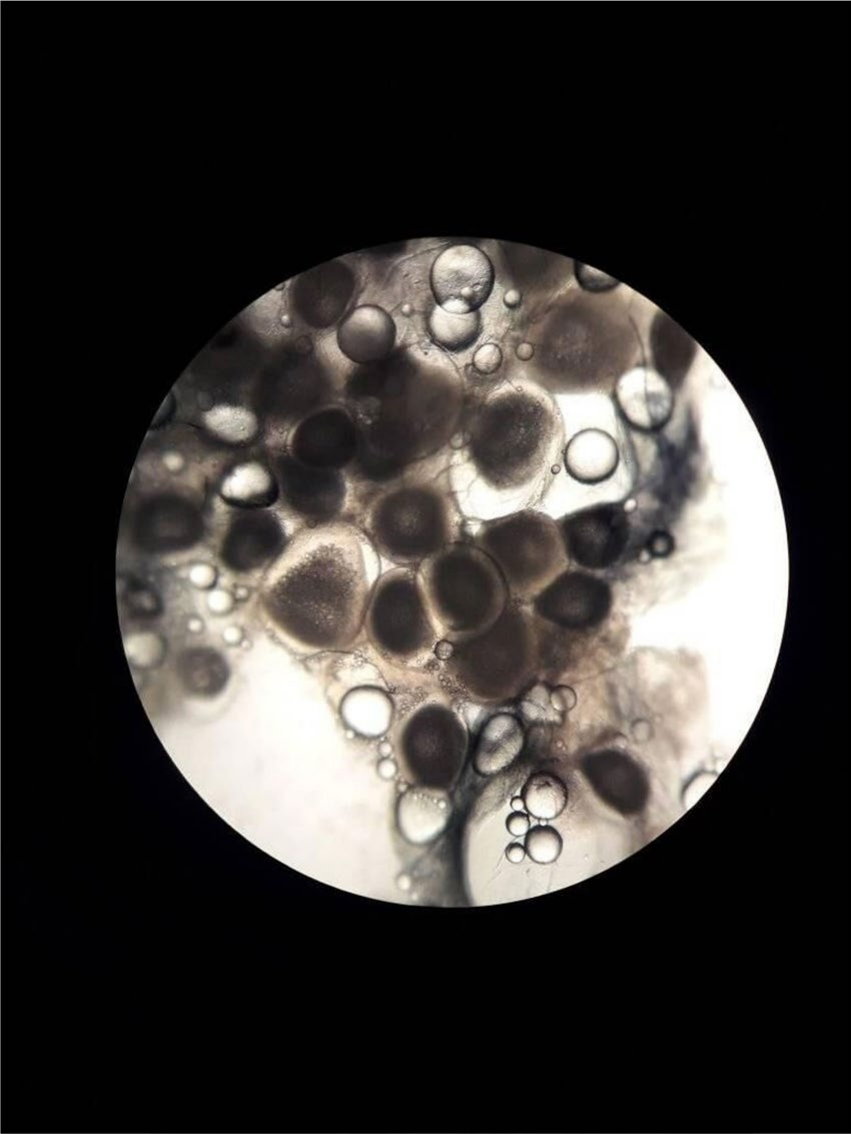
Ovarian sample from the mature eel. Visible are transparent oocytes with few or a single lipid droplet and still-translucent oocytes that just started hydration.

**Figure 2:**
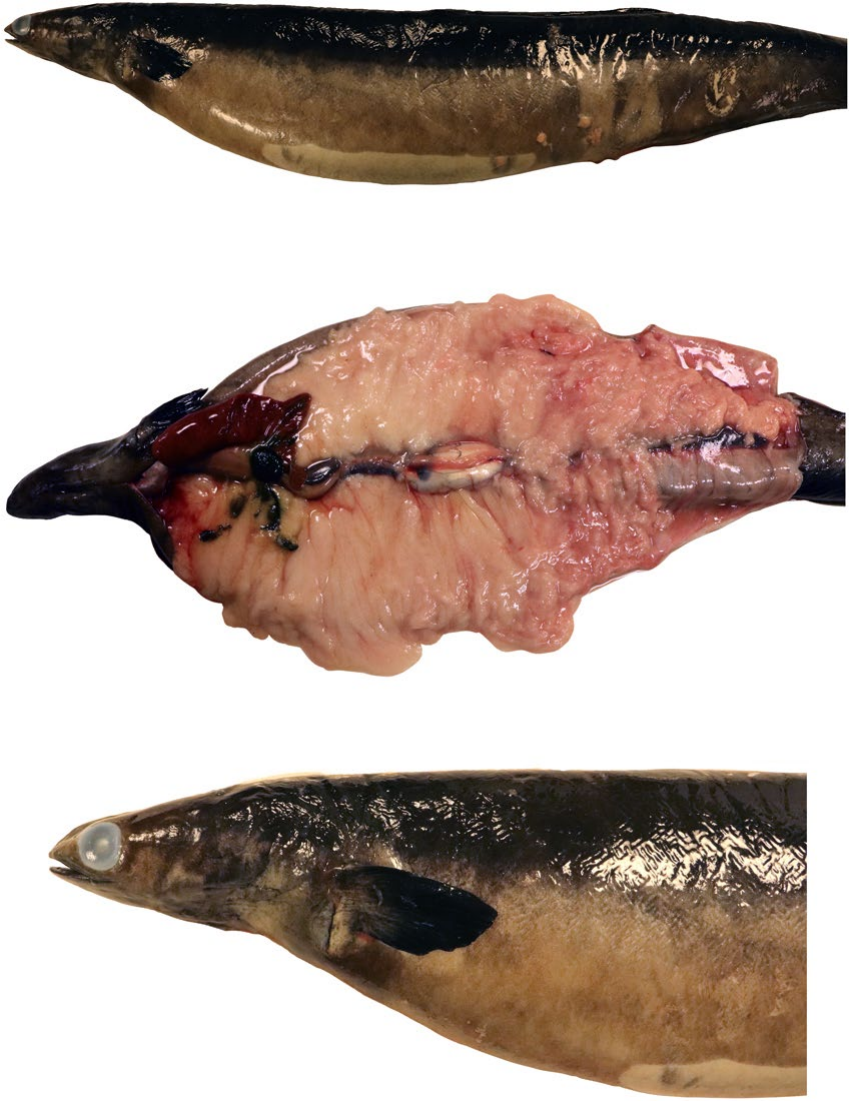
The mature eel with increased belly girth (top), fully developed ovaries (middle) and portrait showing the large eye and dark pectoral fin (bottom).

**Figure 3:**
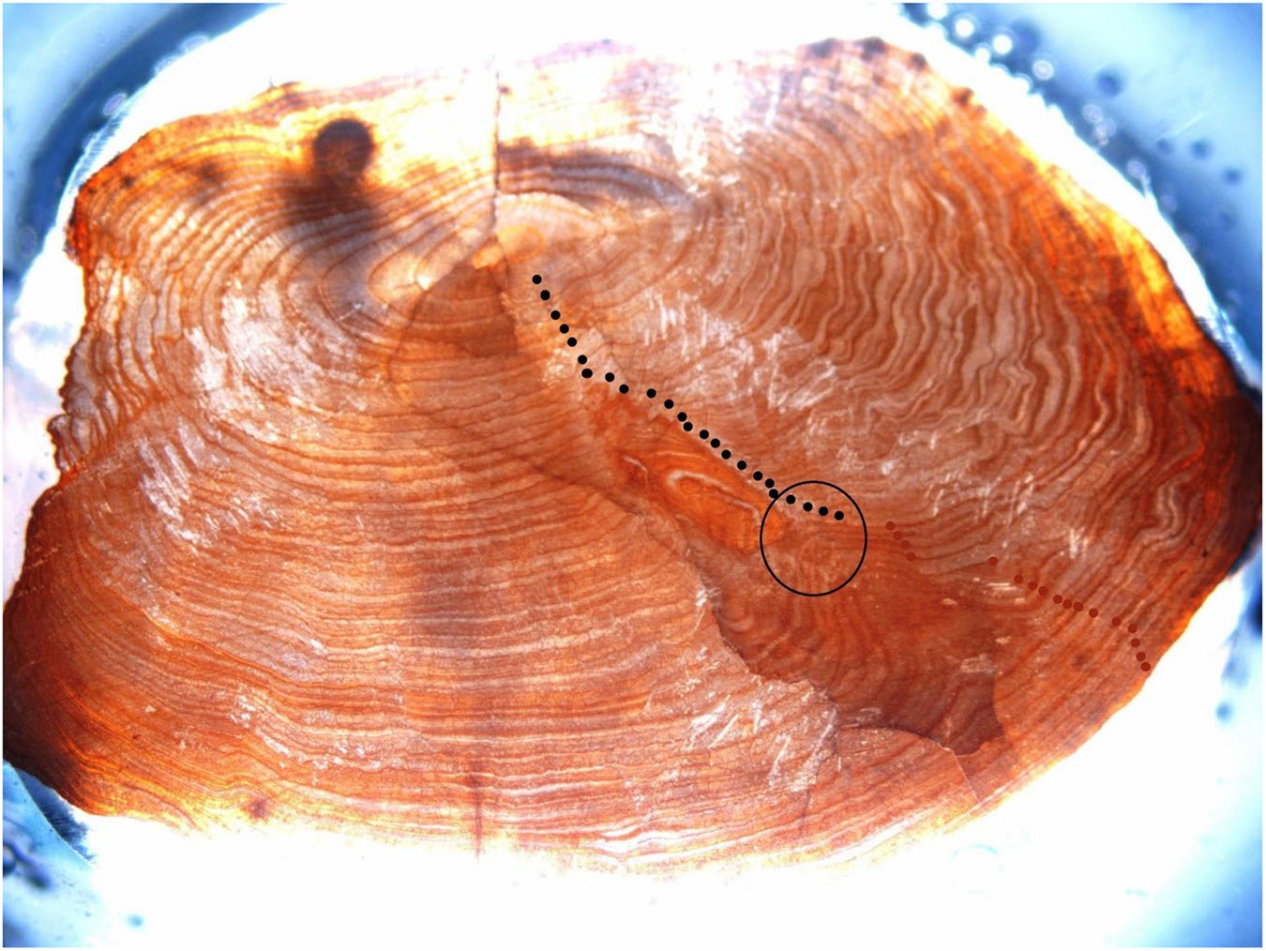
The otolith of the 43 year old matured eel. Indicated and clearly visible is the change in annulus (year ring) structure after the transfer to the Kotka Maretarium in 2002. The annuli in the period before 2002 are marked with black dots (N = 24 confirming the stocking year 1978), and after 2002 with red dots (N = 17 adding up to 2019 with the annulus for 2019 not present yet).

Table 1 summarises the data that were collected. The ovarian samples of the matured eel showed ripe and overripe oocytes with a single lipid droplet but also a second batch of still-translucent oocytes that just started hydration (Fig. 1). The GSI, calculated at 31.2 (Fig. 2), was estimated on the basis of the defrosted body weight (2,587 g) rather than the fresh weight (3,360 g); however, as much of the lost weight may have had an ovarian origin, the ‘true’ GSI is likely to have been higher (up to 47.0 if all loss was derived from gonadal tissue). The advanced sexual maturation status was also illustrated by an eye index of 24.9 and a BGI of 0.271. The otolith annuli matched the number of years in the period from stocking in 1978 to recapture and transfer to the Maretarium in 2002 (24 annuli), and the years in the aquarium thereafter (17 annuli plus one still forming; Fig. 3), adding up to the age of 43 (when a French glass eel age of 1 year is considered^9^). There was no evidence for the presence of the swimbladder parasite *Anguillicoloides crassus*.

**Table 1:** Biometric data of female eels at Maretarium, Kotka, Finland.

		BL	BW	K	BGI	EI	PFI	GSI	HSI	ISI	CSI
matured eel											
*fresh*			3360					47.0			
*defrosted*		112	2587	0.184	0.271	24.9	4.89	31.2	2.05	0.232	0.193
live eels		111	3036	0.222	0.241	21.2	4.85	<2			
		102	2680	0.253	0.223	18.1	5.07	1.6			
		92	1707	0.219	0.215	27.2	5.78	2.0			
		115	4165	0.274	0.212	18.2	4.37	<2			
		94	1773	0.213	0.210	24.4	5.21	1.0			
		104	2285	0.203	0.209	16.7	5.63	1.5			
		104	2418	0.215	0.208	18.7	4.79	1.0			
		104	2090	0.186	0.198	14.2	4.83	1.7			
		114	2656	0.179	0.195	20.2	4.30	1.3			
		110	2586	0.194	0.194	20.8	4.30	0.9			
		117	2717	0.170	0.190	19.7	4.19	1.6			
	**av**	**106**	**2556**	**0.212**	**0.208**	**20.0**	**4.85**	**1.4**			
	**sd**	**8**	**672**	**0.031**	**0.015**	**3.6**	**0.54**	**0.4**			

The fresh carcass of a mature eel was weighed and GSI was estimated by compensating for weight loss (*see text*). Other data from the mature eel were collected from the defrosted carcass. Data from eleven live eels are ordered by BGI, under the assumption that the biggest belly would indicate most advanced gonadal development. GSI values of two eels could not accurately be determined by ultrasound but were smaller than 2. All live eels were still previtellogenic but the eyes were very large. BL = body length; BW = body weight; K = Fulton’s condition factor; BGI = body girth index; EI = eye index; PFI = pectoral fin index; GSI = gonadosomatic index; HSI = hepatosomatic index; ISI = intestinal somatic index; CSI = cardiac somatic index.

### The live eels

All live eels were still in the previtellogenic stage as indicated by the GSI estimated between 0.9 and 2.0 (Table 1), non-invasively determined by ultrasound. The eye indices were ranging from 14.2 up to 27.2, even larger than that of the mature specimen. BGI values were on average 0.208 ± 0.015, one eel reaching a value of 0.241.

## Discussion

This study reports on the first observation of a spontaneously matured female European eel. The eel reached full sexual maturity in June which is late in comparison with the expected spawning time in the Sargasso Sea^2^. The specimen had an estimated maximum GSI of 47 which is within the range (30–60) that we found for artificially matured European eels^10^. This GSI value corresponds to a fecundity of an estimated 3.7 million eggs^11^. Similarly, the eye index and body girth index values were typical for a fully mature eel. About half of the oocytes in a freshly collected sample were hydrated and mature, deemed mostly overripe. The other half consisted of oocytes that just commenced hydration, indicating that European eels are batch spawners and polycyclic like Japanese eels^3^, presumably spawning more than once during a single spawning season.

After a long growth phase as yellow eels, and just before commencing their spawning migration, eels show the first signs of sexual maturation and pre-adaptation to the oceanic conditions: they become silver. European silver eels are typically in a previtellogenic stage with GSI values between 1 and 2.5. Although silver eels are still just at the brink of puberty, this is the most advanced stage of natural sexual maturation we know. Three incidental observations exist of migrating female silver eels in the open ocean. Ernst^12^ reported on a female eel that was caught near the Faroe Islands and that had a GSI of 2.9. One female eel was caught near the Azores and had a GSI of 9.8^13^. Finally, Robins *et al*.^14^ photographed a migrating eel with a distended belly at the Bahamas at 2000-m depth. A fully mature eel in the Sargasso Sea has never been observed making the mature eel from the Maretarium the first observation.

All eels of the live cohort were previtellogenic and presented with the GSI values that are well short from those seen during vitellogenesis (2.5–20) and full maturity (20–60). Generally, the progression of gonadal development is reflected in the eye index but for these eels, the eyes were very large while the GSI was still low (0.9–2.0). As the distended belly of the matured eel was noticed in June, triggering of sexual maturation may have occurred in early spring. Next spring, one or more maturing eels may be expected.

Abiotic conditions that the spontaneously matured female eel experienced were very different from those experienced by female eels during their oceanic migration and in the Sargasso Sea. The exact cue for leaving the European continent and commencing the oceanic migration is still unknown. Eels generally leave in autumn at high levels of water discharge and at water temperatures between 9 and 12 °C (Vøllestad *et al*.^15^). During the early stages of oceanic migration, they exhibit diel vertical migrations from swimming at 200 m depth during night time at 12 °C to descending as far down as 1000 m depth during day time at 10 °C (Aarestrup *et al*.^16^). The collection of recently hatched eel larvae in March and April marks the Sargasso Sea spawning site at 24–30 °N and 71–52 °W across a 2,000 km wide region of the North Atlantic Ocean (Miller *et al*.^17^). Temperature at the hypothesized spawning site is 18 to 22 °C and salinity ranges between 36.4 and 36.8 ppt (Schabetsberger *et al*.^18^). Except for the gradual temperature increase, none of the conditions experienced in Maretarium resemble the oceanic conditions. Therefore, none of the abiotic conditions imposed on the fish during captivity are likely to have acted as candidates for triggering the sexual maturation.

Abiotic conditions showed regular seasonal fluctuation over the year without any clear difference that could explain the sudden triggering of the reproductive axis of the eel. One change was significant, however: the introduction of bream in the tank in autumn. The male bream showed a continuous spawning rash, an androgen-mediated secondary sexual characteristic^19^, ever since their introduction, but there was no evidence of spawning having occurred. We propose that the bream and/or other maturing and spawning fishes in the same aquarium system, have released substances into the water that are responsible for triggering of sexual maturation in the eel. Its old age may have sensitized the eel for such triggers. These substances could be hormones, released as pheromones, and may be steroids or their sulfo- or glucuro-conjugated metabolites^20^. Steroids such as 11-ketotestosterone have a relatively long half-life and can initiate vitellogenesis in eels^21,22^.

With the first European eel having matured spontaneously, and with the potential of more eels that will mature in the near future, finally a natural reference is provided for the sexual maturation of the iconic European eel.

## Supplementary information

Supplementary Information.

## Data Availability

Data are available from the corresponding author upon reasonable request.
