# Supplementary material for: First Observation of a Spontaneously Matured Female European Eel (Anguilla anguilla)
Source: Sci Rep. 2020 Feb 11;10:2339. doi: 10.1038/s41598-020-59331-6 (PMC7012921; doi:10.1038/s41598-020-59331-6)
Supplement: Supplementary file 1 — Supplementary Information. [file 41598_2020_59331_MOESM1_ESM.pdf]

Supplemental material

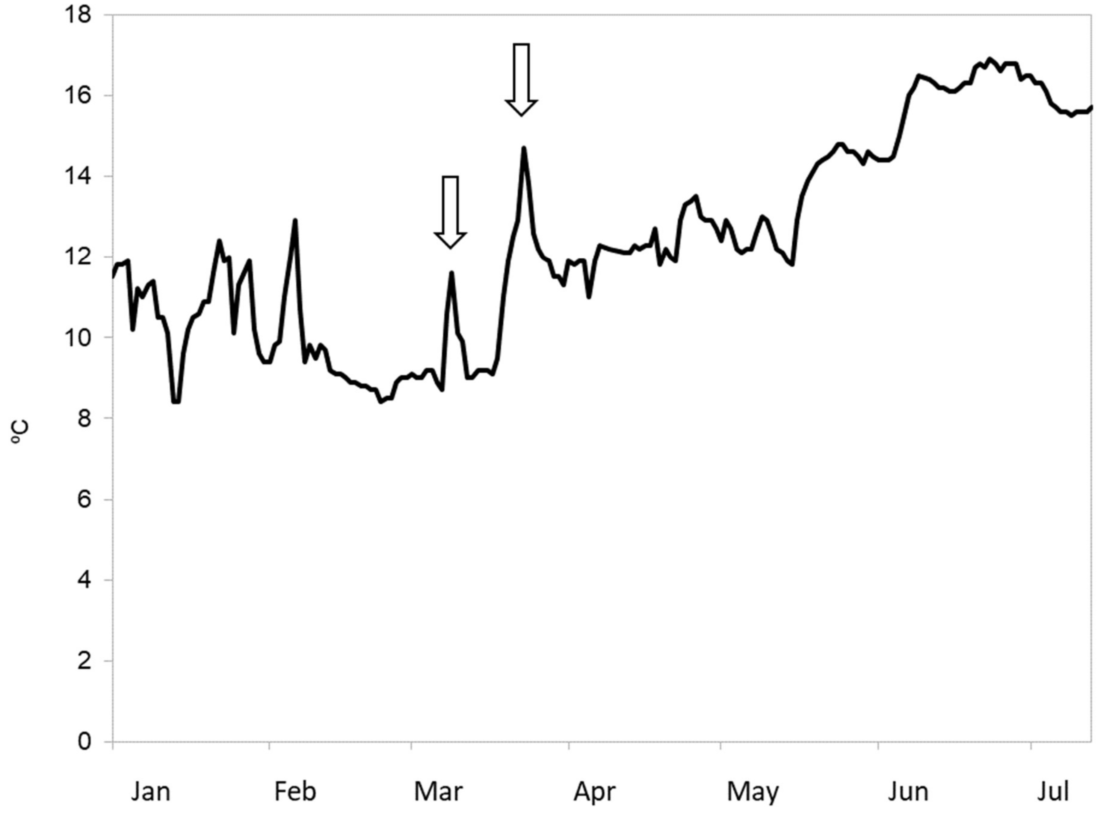

Water temperatures in the period January 1 2019 until the moment of death of the matured female on July 13 2019 increased from 8 to 14 ºC with two small daily peaks occurring in March.
